# Recent HIV infections: evaluation of a simple identification score for newly diagnosed patients

**DOI:** 10.11606/s1518-8787.2022056004047

**Published:** 2022-04-27

**Authors:** Elaine Monteiro Matsuda, Cintia Mayumi Ahagon, Luana Portes Ozório Coelho, Ivana Barros de Campos, Daniela Rodrigues Colpas, Andreia Moreira dos Santos Carmo, Luís Fernando de Macedo Brígido

**Affiliations:** I Secretaria Municipal de Saúde Santo André SP Brasil Secretaria Municipal de Saúde. Santo André, SP, Brasil; II Instituto Adolfo Lutz Centro de Virologia São Paulo SP Brasil Instituto Adolfo Lutz. Centro de Virologia. São Paulo, SP, Brasil; III Instituto Adolfo Lutz Centro Regional de Santo André Santo André SP Brasil Instituto Adolfo Lutz. Centro Regional de Santo André. Santo André, SP, Brasil

**Keywords:** Acquired Immunodeficiency Syndrome, epidemiology, AIDS Serodiagnosis, classification, Early Diagnosis, Time-to-Treatment, Epidemiologic Surveillance Services

## Abstract

**OBJECTIVE:**

Recognize incident infection to better characterize the groups that fuel HIV epidemic. We propose a simple score to identify recent infections among newly diagnosed patients as a HIV surveillance tool.

**METHODS:**

Newly diagnosed patients were defined as recent infections when a negative serological test in the previous year was available. Laboratory tests, such as the avidity index (Bio-Rad, according to the CEPHIA protocol), chemiluminescent intensity (CMIA, architect, Abbott), and the nucleotide ambiguity index of partial *pol* sequences were used as proxies of recency. A simple score based on clinical symptoms of acute retroviral syndrome during the previous year, CD4+ T cell count, and viral load at admission was tested to assess the predictive power, using receiver operating characteristic (ROC) curves, to identify recent cases of infection.

**RESULTS:**

We evaluated 204 recently diagnosed patients who were admitted to the *Ambulatório de Referência em Moléstias Infecciosas de Santo André* (Santo André Reference Infectious Diseases Outpatient Clinic), in the metropolitan region of São Paulo, Brazil, recruited between 2011 and 2018. An HIV-negative test in the year prior to enrollment was documented in 37% of participants. The proportion of cases classified as recent infections (less than one year), according to the laboratory proxies were: 37% (67/181) for an avidity index < 40%, 22% (30/137) for a CMIA < 200, and 68% (124/181) for an ambiguity index < 0.5%. Using different combinations of recency definitions, our score showed an area under the ROC curve from 0.66 to 0.87 to predict recency.

**CONCLUSIONS:**

Using data from patients’ interviews and routine laboratory tests at admission, a simple score may provide information on HIV recency and thus, a proxy for HIV incidence to guide public policies. This simple for the Brazilian public health system and other low- and middle-income countries.

## INTRODUCTION

Forty years into the Aids epidemic, the current number of deaths from the disease is the lowest for the last 20 years (690,000 HIV-related deaths in 2019). However, estimates suggest that 1.5 million new infections still occur annually^1^. The number of new infections fuels the epidemic, which seems to have increased in many areas. Due to different obstacles, added now by the COVID-19 pandemic, which also affected people living with HIV care^2^, most countries will fail to reach the 90-90-90 goal in the near future.

Despite many different interventions, HIV infection in Brazil shows signs of continued expansion. In 1996, the Brazilian government initiated free treatment policies, along with prevention campaigns and free tools, such as condoms, post-exposure prophylaxis and, more recently, pre-exposure prophylaxis, but all these initiatives have been insufficient to restrain the epidemic. Brazil has had an estimated 40,000 new HIV cases annually in the last five years^5^, with increasing trends in some populations, such as men who have sex with men^6^. Still, information on the incidence of HIV infection is limited.

Viral load testing of seronegative individuals with recent risk of exposure is the most reliable way to identify a very early (acute) infection, and, despite proposals of clinical algorithms to screen eligible patients^7^, costs and logistic issues limits routine use. Identifying incident cases with information available at routine admissions to health services in low- and middle-income countries may provide a practical and useful tool to help public health policies to target segments of the population which are more vulnerable to HIV infection, thus improving diagnosis, linkage to care, and providing epidemiological information relevant to the understanding of the HIV epidemic.

This study aims to evaluate laboratory proxies to identify recently infected HIV patients and to assess a simple score to predict recency based on information available at admission, a tool that could be integrated into the case notification of HIV surveillance services without additional costs, especially in low- and middle-income countries where resources are scarce.

## METHODS

### Patients and Methods

Participants were enrolled among newly diagnosed patients, recruited from 2011 to 2018. Patients were informed of our study and included if had a first HIV positive test recently and agreed to participate in the study, signing an informed consent form. Data from the Brazilian surveillance system were also included. This study was registered and approved by institutional ethics committees (CAAE: 39967314.5.0000.5484 and CAAE: 39967314.5.3001.0059).

Participants were included in our analyses if they met one of the following criteria: i) patients recently diagnosed by serological testing (both recent and chronic HIV infections), admitted to the *Ambulatório de Referência em Moléstias Infecciosas de Santo André*, in the metropolitan region of São Paulo, Brazil; ii) patients with acute HIV infections diagnosed by viral load (> 5,000 copies/mL), and lacking criteria for the serological diagnosis of HIV infection; and iii) patients evaluated for the clinical symptoms of dengue infection lacking its serological markers, but with a detected HIV viral load and negative or indeterminate HIV serological results^8^.

To exclude patients already in follow-up, cases were further validated as actual new HIV diagnoses by a review of public health electronic databases which verified the absence of previous antiretroviral registries at either the *Sistema de Controle Logístico de Medicamentos* (the Brazilian drug logistics management system - SICLOM), or at the viral load and CD4+ T cell count registries at the *Sistema de Controle de Exames Laboratoriais* (Laboratory Examinations Control System - SISCEL). CD4+ T cell count was performed by ﬂow cytometry (BD, USA), and HIV viral load by reverse transcription-quantitative polymerase chain reaction (RT-qPCR) (Abbott, USA) at accredited laboratories of the national HIV laboratory network.

To estimate the time of HIV infection, especially for cases without a previous seronegative test, three assays were used; i) the avidity index, based on the commercially available ELISA kit (Genetic Systems HIV-1/HIV-2 Plus EIA, Bio-Rad, USA) performed with modifications based on the DEA-EIA, CEPHIA protocol^9^. Briefly, 100 μL of serum were diluted in an incubation buffer at a 1:10 ratio and incubated in the presence or absence (in parallel) of 0.1 M diethylamine (DEA). The optical density pairs (OD 450nm) of the samples (with and without DEA) were used to estimate the avidity index according to the following equation: avidity index % = [OD 450nm (with DEA) / OD 450 nm (without DEA)] × 100. Two cases of acute infection with no change in OD after modification due to a lack of reactivity even in regular serological tests were classified, for this study, as an avidity index of 0%. A second serological proxy for recency was the chemiluminescent microparticle immunoassay (CMIA), its values were generated by the architect microparticle based immunoassay (Abbott USA), a fourth-generation serological test, performed according to the manufacturers’ instructions. Time of infection was additionally estimated by the sequence ambiguity index, based on partial polymerase sequences. Briefly, sequences were generated from retrotranscribed plasma RNA by one-step RT-PCR using High Fidelity Taq platinum and Superscript III, followed by nested PCR and Big Dye (Life) incorporation, resolved in an ABI 3130XL^10^. Sequences were edited with the Sequencer 4.7 software (Gene Codes) and/or with Recall (http://pssm.cfenet.ubc.ca/), subsequently analyzed at NCBI (www.ncbi.nlm.nih.gov/projects/genotyping/help.html) and REGA Genotyping tools (http://www.bioafrica.net/rega-genotype/html/). To estimate the ambiguity index, the number of positions with possible nucleotide mixtures observed in the sequence was divided by the total number of nucleotides and multiplied by 100. The unresolved mix of all nucleotides (N) was excluded from both the denominator and the numerator of the calculation. The percentages of the ambiguous bases (R, Y, K, M, S, W, B, D, H, V) in each sequence were estimated with BioEdit^11^ and Excel softwares.

The recency cutoffs used were as suggested by the literature, such as defining recent infections by an avidity index below 40%^9^ and a cut-off below 0.5% of the ambiguous positions^12^. For CMIA, although a signal/cut-off (S/CO) limit of 400^13^ or 418 (IQR25–75 384–449)^14^ units was used by some studies as a proxy for recent infection, a stringent cutoff of an S/CO below 200 units^15^ was chosen.

To define chronic cases, less stringent definitions were used, such as including all non-acute volunteers without a negative serology in the year prior to sampling, as well as considering chronic cases with only some laboratory evidence of chronic infection, such as a sequence ambiguity above 0.5%, an avidity index above 80%^16^, or a high CMIA. Since the literature lacks a clear value to define what a high CMIA is, a more conservative definition was assumed based on our tested cases, in which the cutoff was defined as a CMIA value above the median of volunteers lacking previous negative test, i.e., 821 units. This value is above the 400 units used in some previous studies as a proxy for chronic cases^13,14^.

### A Score to Identify Recent Infections

Simple clinical and epidemiological criteria available at admission as part of routine patient enrolment into care were given points, resulting in a score to access the likelihood of recent infections:

- Viral load (VL) at admission (VL log_10_ ≥ 5 to 5.9: score +5; VL log_10_ ≥ 6 to 6.9: score +6, and VL log_10_ ≥ 7: score +7).
- CD4+ T cell count at admission (CD4+ < 100 cells/mm^3^: score -7; CD4 + 100 to 199 cells/mm^3^: score -5; CD4+ 200 to 349 cells/mm^3^: score -3, and CD4+ ≥ 350 cells /mm^3^: score 0).
- Clinical history of symptoms in the 365 days prior to diagnosis suggestive of acute retroviral syndrome (ARS): score of +7 if fever accompanied by at least two of the following symptoms: headache, myalgia, arthralgia, rash, oral or genital ulcers, malaise, diarrhea, and lymphadenopathy.
- Unprotected sex with HIV-positive individuals or unknown serological status in the last year: score +10.

### Definitions of Recent Infections

Different associations of three laboratory proxies of infection time; serological (CMIA and avidity) and molecular (ambiguity) were tested with the proposed score. These laboratory proxies were considered by themselves and with the documented, serological evidence of seroconversion in the year prior to sampling.

### Statistical Analysis

Continuous variables were shown as the median and interquartile ranges (IQR), and categorical variables, as proportions. To assess the recency of infection among this population, alternative definitions were tested to categorize cases as recent or chronic infections according to laboratory recency tests and seroconversion data. ROC curves (Stata version 10) were used to determine the best score to discriminate recent infections and evaluate the sensitivity and specificity of the score regarding our definitions. Spearman correlation (Stata version 10) was used to measures the strength and direction of correlation between continuous variables.

## RESULTS

We included 204 newly diagnosed cases. Table 1 shows the demographic and laboratory data of all cases at admission.

**Table 1:** Demographic and laboratory data of the studied population.

Age (years) (n = 204)	27 (IQR25–75: 23–34)
Men	87% 178/204
MSM	81% 145/178
CD4+ T cells/mm^3^ (n = 199)	528 (IQR25–75: 353–769)
Viral load (log_10_) (n = 203)	4.67 (IQR25–75: 4.06–5.19)
Avidity index % (n = 181)	73% (IQR25–75: 21–100)
Avidity index < 40%	37% 67/181
CMIA (n = 137)	580 (IQR25–75: 250–899)
CMIA % of cases with index < 200	22% 30/137
Ambiguity index (n = 181)	0.20% (IQR25–75: 0.00–0.59)
Ambiguity index < 0.5%	68% 124/181
Subtype *pol* (NCBI/REGA) (n = 181)	B 75.7%, C 10.5%, F 5.5%, Rec 8.3%

MSM: men who have sex with men; Avidity index: Bio-Rad-Avidity assay serology test (CEPHIA protocol). CMIA: chemiluminescence microparticle immunoassay (Architect HIV Ag/Ab, Abbott, USA). Ambiguity index: the number of nucleotide mixtures (excluding four unresolved nucleotides) divided by the total number of nucleotides analyzed. Rec: recombinants at partial *pol* (4 AG, 3 BC, and 8 BF). Continuous variables are shown as median and percentile 25th-75th (IQR), and categorical variables, as proportions.

We evaluated associations of the three proxies of recency, with a Spearman’s rank correlation varying from 0.65 rho for the two serological assays (avidity and CMIA indices) to 0.46 rho for avidity and ambiguity indices (p < 0.0001).

### Definitions Used to Classify Cases as Recent or Chronic

In total, 37% of our cases showed a negative test for seroconversion within one year (SC1year), the gold standard for recent infections. Thus, most newly diagnosed cases lacked previous serology, therefore potentially including either untested recent infections or chronic cases. To evaluate if we could use these laboratory proxies to identify additional recent infections among these patients, we first compared the values of these laboratory proxies of recency for cases with one-year seroconversion to all others. This comparison shows a significant difference: CMIA, 251 (IQR25–75 : 98–557) vs 729 (IQR25–75 : 507–1,045) (p < 0.0001); avidity index, 24% (IQR25–75 : 11–77) vs 91% (IQR25–75 : 40–100) (p < 0.0001), and ambiguity index, 0.09 (IQR25–75 : 0–0.3) vs 0.37 (IQR25–75 : 0.09–0.64) (p = 0.0005). Assuming that these proxies could identify additional recently infected individuals, we constructed different recent/chronic definitions to evaluate the consistency of the proposed score.

The area under the ROC curve (AUC) varied from 0.66 (using only the ambiguity index) to 0.87 for the serological indices. Table 2 shows some of the definitions and respective AUC values obtained.

**Table 2:** The area under the ROC curve (AUC) of different definitions evaluated.

	n	AUC	95%CI
SC1YEAR vs all others	204	0.79	0.72–0.87
SC1YEAR vs avidity > 80%	118	0.84	0.76–0.91
SC1YEAR vs avidity > 80% or CMIA > 821	125	0.84	0.77–0.91
SC1YEAR vs either avidity > 80%, CMIA > 821, or ambiguity > 0.5%, except if avidity < 40% or CMIA < 200 (Definition A)	133	0.85	0.78–0.92
SC1YEAR vs avidity > 80% & CMIA > 821	76	0.84	0.75–0.93
SC1YEAR vs avidity > 80% & CMIA > 821 or CDC93 C (Aids)	86	0.87	0.80–0.94
SC1YEAR & avidity < 40% or CMIA < 200 vs avidity > 80% or CMIA > 821	170	0.76	0.69–0.83
SC1YEAR or avidity < 40% or CMIA < 200 vs avidity > 80% or CMIA > 821	182	0.78	0.71–0.84
Ambiguity - low (< 0.5) vs high (> 0.5)	181	0.66	0.58–0.75
Avidity - low (< 40%) vs high (> 80%)	147	0.71	0.63–0.79
Avidity < 40% vs avidity > 40%	181	0.74	0.64–0.84
CMIA < 200 vs CMIA > 821	72	0.77	0.66–0.88
CMIA < 200 vs CMIA > 400	121	0.71	0.61–0.81
CMIA < 200 vs CMIA > 200	137	0.71	0.60–0.81
CMIA < 400 vs CMIA > 400	137	0.74	0.66–0.83
CMIA < 400 vs CMIA > 821	88	0.78	0.68–0.87

AUC: the area under the ROC curve. SC1YEAR: documented one-year seroconversion. Vs: versus. CDC93: Center of disease control 1993 HIV infection staging classification. Avidity index: Bio-Rad-Avidity assay serology test (CEPHIA protocol). CMIA: chemiluminescence microparticle immunoassay Architect HIV Ag/Ab, Abbott, USA). Ambiguity index: the number of nucleotide mixtures (excluding four unresolved nucleotides) divided by the total number of nucleotides analyze. 95%CI: confidence interval of the AUC generated at Stata version 10.

The definition of recent and chronic infections that had the best area under the curve (0.87 AUC), but includes clinical information such as chronic infection classification and has a smaller sample size (n = 86). So we adopted the second-best AUC, 0.85 (definition A). This definition compares SC1year (recent) to chronic cases defined as either an avidity > 80%, a CMIA > 821, or an ambiguity > 0.5, except if avidity < 40% or CMIA < 200 (n = 133). This definition includes as chronic all three laboratory cutoffs but excludes any case with one of the proxies of recency. Table 3 shows the sensitivity and specificity (ROC) curve of the score with this adopted definition (A), and Figure, its respective ROC graph.

**Table 3:** Sensitivity, specificity and other parameters of ROC curve for the score obtained with definition A. Detailed report of sensitivity and specificity.

Correctly					
Cut point	Sensitivity	Specificity	Classified	LR+	LR-
≥ 3	100.00%	0.00%	31.58%	1.0000	
≥ 7	100.00%	4.40%	34.59%	1.0460	0.0000
≥ 8	100.00%	7.69%	36.84%	1.0833	0.0000
≥ 9	100.00%	21.98%	46.62%	1.2817	0.0000
≥ 10	100.00%	25.27%	48.87%	1.3382	0.0000
≥ 12	83.33%	68.13%	72.93%	2.6149	0.2446
≥ 13	83.33%	73.63%	76.69%	3.1597	0.2264
≥ 15	83.33%	78.02%	79.70%	3.7917	0.2136
≥ 16	69.05%	85.71%	80.45%	4.8333	0.3611
≥ 17	66.67%	85.71%	79.70%	4.6667	0.3889
≥ 20	42.86%	94.51%	78.20%	7.8000	0.6047
≥ 21	40.48%	95.60%	78.20%	9.2083	0.6226
≥ 22	38.10%	95.60%	77.44%	8.6667	0.6475
≥ 23	11.90%	100.00%	72.18%	0.8810	
≥ 24	7.14%	100.00%	70.68%		0.9286
> 24	0.00%	100.00%	68.42%		1.0000
**ROC**			**Asymptotic Normal**		
**Obs**	**Area**	**Std. Err.**	**[95% Conf.**	**Interval]**	
133	0.8503	0.0343	0.78311	0.91757	

Definition A: only recent cases with documented seroconversion within one year (SC1year) versus either the avidity index > 80% (Bio-Rad-Avidity assay serology test, CEPHIA protocol), CMIA > 821 (chemiluminescence microparticle immunoassay Architect HIV Ag/Ab, Abbott, USA), ambiguity index > 0.5% (the number of nucleotide mixtures, excluding four unresolved nucleotides divided by the total number of nucleotides analyzed), except if avidity index < 40% or CMIA < 200.

**Figure:**
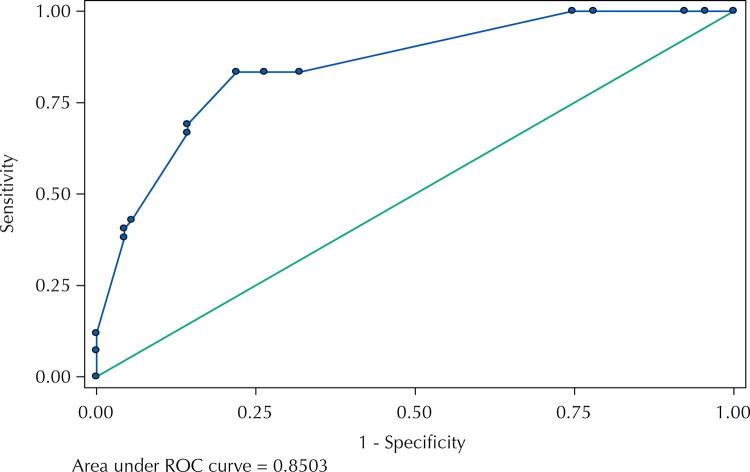
The ROC graph of the score for definition A.

## DISCUSSION

Serious challenges remain in addressing the global HIV/Aids pandemic, so it is imperative that we maximally implement the effective prevention and treatment tools already available to us^12^. Among these are instruments to better identify the source of new infections in the universe of prevalent cases, fundamental to the better understanding of HIV incidence. Identifying incident/recent infections has been a challenge, but it is a key metric for epidemiological monitoring and intervention. Therefore, estimating the incidence or the number of new infections at a certain time is a major tool for preventive interventions^17^.

Cohorts of seroincidence are costly, and inclusion and retention biases may distort estimates. A large CEPHIA consortium has concatenated different efforts in the field to provide tests that may evaluate the recency of HIV infections in samples with known time of infection. Many of these approaches use modified serological tests, such as the avidity index^15,18^, and, more recently, results from regular serological assays, such as the fourth-generation serological test (CMIA, Architect Abbott, USA), which can provide a dynamic range of chemiluminescence that allows recency estimation^15^. Molecular methodologies, including the percentage of nucleotide ambiguity in viral sequences, as other proposals^12^, are expensive and generally unavailable in routine workups. The immediate treatment policy^19,20^ will further limit the use of serological tests to classify HIV recency in future samples, as antiretroviral drugs (ARVs) may modify the maturation of serological responses^21,22^.

We evaluated this small cohort of newly diagnosed cases to characterize the time of infection using three laboratory proxies of infection time (CMIA, avidity and ambiguity indices) from known, well-characterized, untreated patients. These measures were highly correlated, suggesting their usefulness, individually or associated to improve specificity.

As a gold standard for defining recency, we used the results of negative HIV tests in the year prior to enrollment, but only 37% (75/204) of individuals had this information. Reports, or even documentation of previous serological tests, do not guarantee actual seronegative status as they depend on the quality of the information provided by volunteers (a problem for self-reported seroconversion), as well as potential false-negative point-of-care tests^23^, a possibility for both reported and documented tests. However, even considering other biological causes of false negativity, such as during the eclipse or early Fiebig stages in very recent infections, this information, when available, may allow the estimation of the duration of infection^24^.

As many patients do not repeat HIV tests regularly, we use the three laboratory proxies performed (CMIA, avidity and ambiguity indices) to define recent and chronic cases. A simple tool for measuring the incidence of HIV infection has been a goal of public health authorities for a long time^25^. As many cases^23^ report information compatible with the acute retroviral syndrome during the admission routine and laboratory parameters (CD4+ T cell count and viral load) are generally available, this information could help to discriminate between recent and chronic cases. Thus, we proposed and evaluated a score to identify incident cases which is applicable to environments with limited resources.

The probability of a recent infection decreases with CD4+ T cell counts. CD4+ T cell count based incidence models predict, on average, 3–4 years until CD4+ T cells drop below 350^26^. So, we assigned negative score points to cases in which CD4+ T cells counts were below 350 cells/mm^3^. Seroconversion studies show that the highest viremia values occur about one week after the onset of symptoms^23,24^. Thus, we attributed more points to higher viremias. We combined these parameters, available in many HIV surveillance systems, along with risk exposure, to score the likelihood of a recent infection.

Several countries have used models based on CD4+ T cell values close to diagnosis to estimate the number of incident infections. Brazil has a national information system (SISCEL) that monitors CD4+ T cells and HIV viral load data to evaluate patients’ treatment with antiretroviral therapy. The system is considered complete because it is based on government reimbursement. However, SISCEL lacks tests in the private sector (estimated to be 28%)^25^. Including this information in notification forms and linking the prescription of antiretroviral drugs to notification would possibly contribute to expanding the knowledge of public health systems on HIV incidence.

We assessed this score with different definitions of recency and chronicity and found comparable results, suggesting it could provide a useful instrument to monitor the HIV epidemic, an example that additional tools can improve and complement. This pilot study needs validation with a larger and diverse populations. Our proposal fails to identify all incident cases. However, implementing a score for surveillance may prove useful. It is nevertheless also important to continue to improve surveillance by incorporating additional tools once they become available, such as the commercially available Asante test (http://www.sediabio.com/products/asante-rapid-hiv-1-recency-assay), a rapid point-of-care test that has included a recency band^26^. As point-of-care viral load testing is increasingly used; these and other tools may allow a more precise understanding of the recency of all new HIV diagnoses tested at HIV-testing services, forming the basis of real-time surveillance.

This study has many limitations, such as its small number of subjects, the fact that most cases involve male homosexuals from a same geographical area, as well as the lack of samples from some cases to perform all three recency tests. However, the fact that the three tests reasonably correlated to each other allowed the classification of cases based on available tests. Recognizing incident infections is relevant to prioritize public policies which would direct resources to prevention and diagnosis campaigns in key populations. These initiatives may help programs to improve epidemiological metrics and focus interventions on those that need them the most^17^.

## CONCLUSION

An easy-to-apply score with information available at routine admission of healthcare centers allowed us to identify recent infections, which could contribute to identifying incident cases and may be incorporated in the surveillance forms (surveillance of HIV/Aids infections) at no additional cost. A better understanding of the dynamics of the HIV epidemic would allow public health systems to prioritize prevention interventions to populations at greater risk of acquiring the infection, providing an additional surveillance metric that may promote early diagnosis, link to treatment and viral suppression, with benefits for individuals’ health and the community.
